# Reversible intramolecular photocycloaddition of a bis(9-anthrylbutadienyl)paracyclophane – an inverse photochromic system. (Photoactive cyclophanes 5)

**DOI:** 10.3762/bjoc.5.20

**Published:** 2009-05-07

**Authors:** Henning Hopf, Christian Beck, Jean-Pierre Desvergne, Henri Bouas-Laurent, Peter G Jones, Ludger Ernst

**Affiliations:** 1 Institut für Organische Chemie, Technische Universität Braunschweig, Postfach 3329, D-38023 Braunschweig, Germany; 2 ISM, CNRS UMR 5255, Université Bordeaux 1, F-33405 Talence Cedex, France; 3 Institut für Anorganische Chemie, Technische Universität Braunschweig, Postfach 3329, D-38023 Braunschweig, Germany; 4 NMR-Laboratorium der Chemischen Institute der Technischen Universität Braunschweig, Hagenring 30, D-38106 Braunschweig, Germany

**Keywords:** anthracenes, cyclophanes, inverse photochromism, photocycloaddition

## Abstract

The title compound, 4,13-bis[(1*E*,3*E*)-4-(9-anthracenyl)buta-1,3-dienyl][2.2]paracyclophane (**2**), prepared in 35% overall yield from [2.2]paracyclophane, absorbs light at λ_max_ = 400 nm with a tail down to 480 nm. By irradiation into this band, **2** generates a single photoproduct, **4**, whose absorption maximum is situated at 306 nm. The starting material is recovered by irradiation at 306 nm or by heating. This ‘inverse’ photochromic system has a potential for optical information storage, compound **4** being stable in visible light, at ambient temperature.

## Introduction

**Figure 1:**
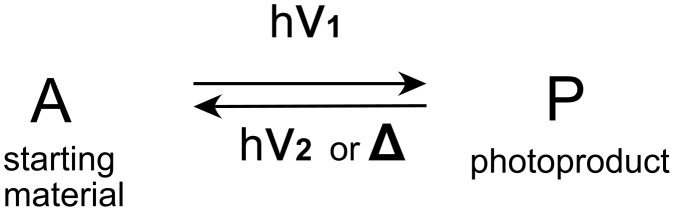
Schematic representation of a photochromic system. The reverse reaction can be a photochemical or thermal process. A and P have different absorption spectra.

Photochromism [1], see Figure 1, is currently an active field of research as reflected in the literature [2–6] and finds commercial applications in the domain of reversible optical density materials. Diverse systems are under study for information storage and optical switches [7–9]. The majority of established systems show a ‘positive’ photochromism in which the electronic absorption spectrum of the product P is red-shifted [1]. When the electronic absorption spectrum of P is blue-shifted, the photochromism is said to be ‘negative’ or ‘inverse’ [1].

**Figure 2:**
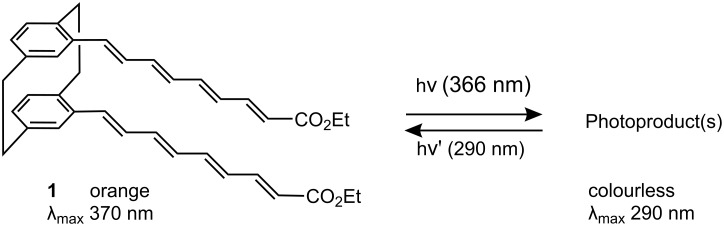
Photochromic reaction of pseudo-gem disubstituted tetraene [2.2]cyclophane **1** in acetonitrile, conc. 5 × 10^−4^ M.

Inverse photochromism is encountered in photoaddition of conjugated systems [10–17] and may be interesting for information storage materials because the photoproducts can be operated in visible light. Inverse photochromism was recently observed for some vinylogs of cinnamophanes [18–19], such as compound **1** (Figure 2). Several cycles were recorded but the photoproduct could not so far be isolated.

**Figure 3:**
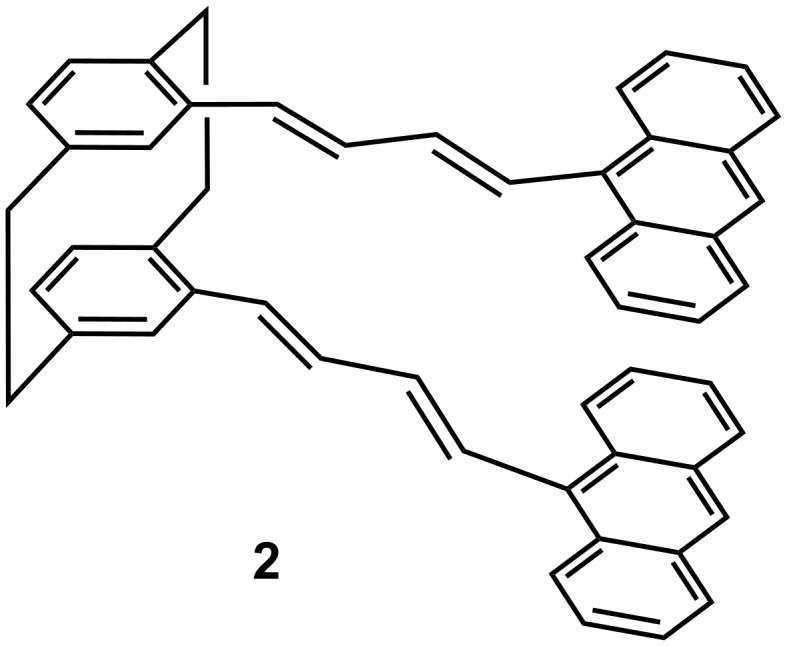
Molecular structure of 4,13-bis[(1*E*,3*E*)-4-(9-anthracenyl)-buta-1,3-dienyl][2.2]paracyclophane (**2**).

It occurred to us that the performance of these systems could be improved by incorporating the anthracene substrate, well known for its ability to generate definite photodimers [10–15], in the pseudo-gem positions of the cyclophane acting as a convenient scaffold, as demonstrated previously [18–19]. To avoid steric crowding and excessive distance between the chromophores, it seemed reasonable to select the rigid all-*trans* butadienyl tether as represented in compound **2** (see Figure 3).

The synthesis, molecular structure and photochromic properties of **2** are reported in this paper.

## Results and Discussion

### 1 Synthesis

**Scheme 1:**
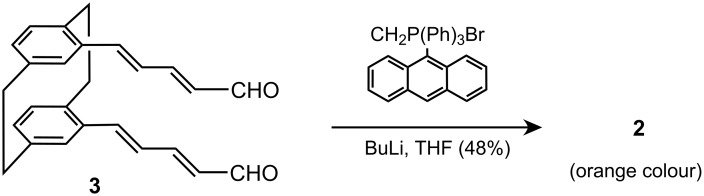
Preparation of **2** (last step), using the Wittig reaction. The preparation of **3** has been described in ref [19].

Compound **2** was prepared from dialdehyde **3** using the Wittig reaction, as outlined in Scheme 1, in 48% yield. Dialdehyde **3** wasobtained in five steps from [2.2]paracylophane, a commercial product, in 72% overall yield as described previously [19]. Therefore, the overall yield of **2** from the parent cyclophane was found tobe 35% on the gram scale.

The Wittig reaction provides a mixture of *cis* and *trans* isomers, but the pure all-*trans* isomer was isolated by crystallization as orange crystals (see Experimental). 9-(anthrylmethyl)triphenylphosphonium bromide was easily obtained from triphenylphosphine and 9-bromomethylanthracene.

### 2 X-ray structure analysis

**Figure 4:**
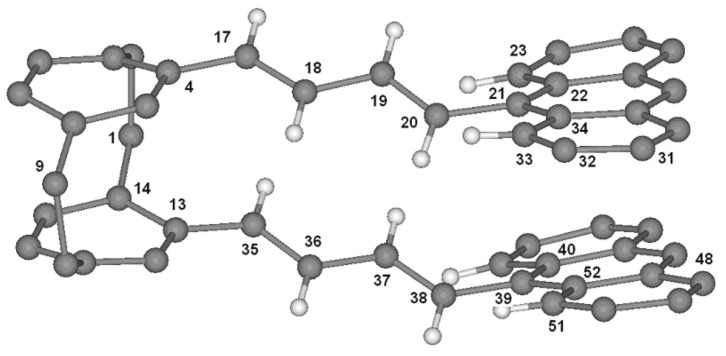
Molecular structure of **2** in the crystal. Radii are arbitrary; only selected H atoms are shown.

Single crystals suitable for X-ray structure determination were grown by slow evaporation of a solution of **2** (150 mg) in dichloromethane (10 mL) by diffusion of pentane vapour. The molecular structure of **2** is presented in Figure 4.

**Figure 5:**
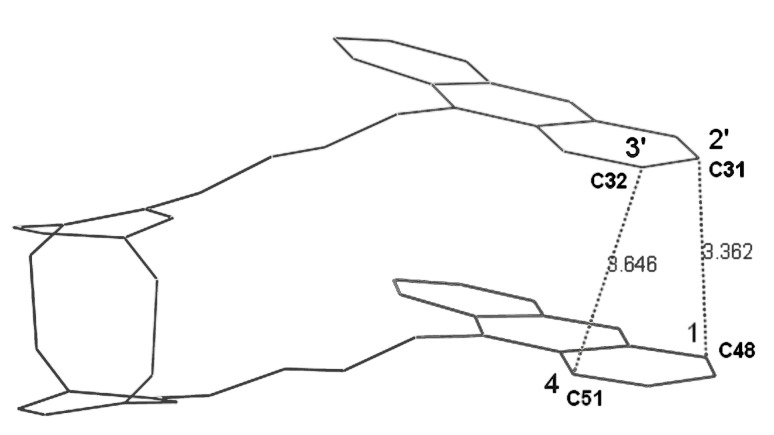
Projection of the molecular structure of **2** exhibiting the closest internuclear distances (distances in Å).

The double bonds are clearly all-*trans* and their average planes form twist angles with the two anthracene nuclei of 47° and 44°,respectively; the two ethylenic systems are also not parallel to each other but subtend twist angles of 42–43° (see Table 1). Finally, the interplanar angle between the two anthracene substrates is ca 4.5° (Table 1). Consequently, the inter-ring distance varies between 3.40 and 3.80 Å. Two lateral anthracene nuclei are thus in close proximity (see Figure 5), in which two carbons are separated by the van der Waals distance, C31-C48 (ca. 3.40 Å); two other carbons are also very close to each other: C32-C51 (ca. 3.65 Å). These remarkable features suggest preferred sites of reactivity inasmuch as this rigid geometry is not disfavoured in solution.

**Table 1:** Angles between various partial planes in molecule **2** in the crystal.

torsion angles	C19–C20–C21–C22	C37–C38–C39–C40
	47.1°	43.9°
interplanar angles	C17–C20/C21–C34	C35–C38/C39–C52
	41.6°	43.3°
deviation from parallelism	butadienes C17–C20//C35–C38	anthracenes C21–C34//C39–C52
	9°	4.5°

### 3 Electronic absorption spectra and photochemistry

#### 3.1 Electronic absorption spectra

**Figure 6:**
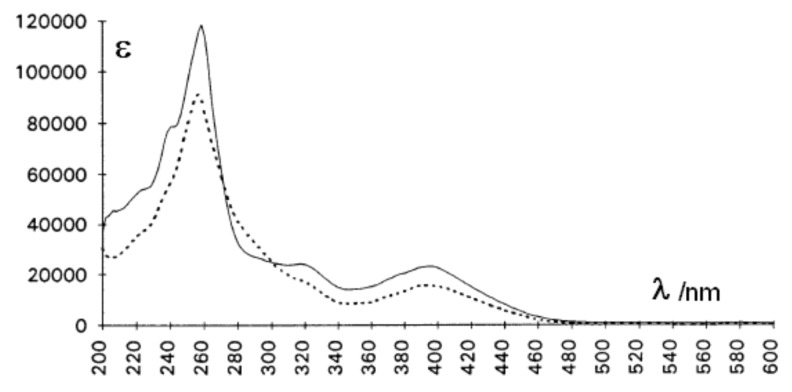
Electronic absorption spectra of **2** (conc. ca 10^−4^ M) in MCH (full line) and CH_3_CN (dotted line) at 20 °C.

The electronic absorption spectra of **2** in methylcyclohexane (MCH) and acetonitrile are represented in Figure 6. One notes that the lowest energy band (λ_max_ = 400 nm with a tail down to 480 nm) is clearly red-shifted as compared to those of all the cinnamophane vinylogs studied so far [18–19], including compound **1** (λ_max_ = 370 nm). The presence of the twisted butadienyl system introduces several torsional vibrations about the pseudo single bonds and attenuates the fine structure usually observed for anthracene derivatives (see Figure 6). The difference between the two solutions is thought to reflect some charge transfer in the more polar solvent, affecting the intensity balance but not the wavelength maxima.

#### 3.2 Irradiation

**Figure 7:**
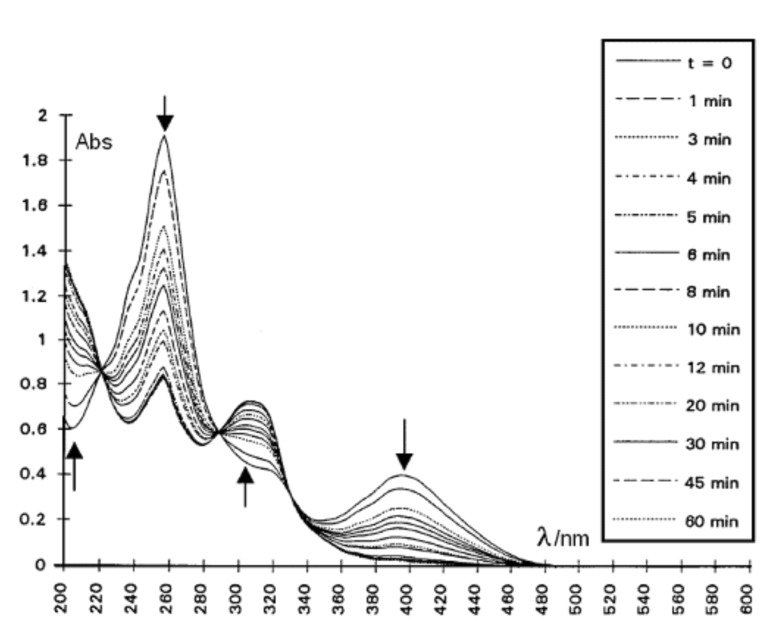
Irradiation of **2** (2.6 × 10^−5^ M) in CH_3_CN at 400 nm at 20 °C. The spectra were recorded at various time intervals within 60 min. The arrows point to the maxima.

Compound **2** was irradiated in acetonitrile in a quartz cell, at 400 nm (selected with a monochromator) using a Xenon lamp. Nitrogen was bubbled through the solution to prevent photooxidation, and the spectra were recorded at various time intervals (Figure 7). Three isosbestic points at 220, 289, and 329 nm were observed as well as new absorptions at 306 nm and in the far UV. Similar features were noted for the MCH solutions. At that concentration, no intermolecular reaction could compete with an intramolecular process [12].

Disappearance quantum yields at 394 nm were found to be low in methylcyclohexane (1.5 × 10^−4^) and in acetonitrile (4.2 × 10^−2^). The weakness of the cyclophotoaddition probably reflects a preference for internal conversion among the diverse deactivation channels of the S_1_ state. The clear difference between the two media may result from an electron transfer between the two anthracene nuclei in the more polar solvent, as demonstrated in previous work [20]. The ion pair can accelerate a closure reaction involving a donor and an acceptor partner, such as a Diels-Alder reaction (see below).

#### 3.3 Photodissociation

**Figure 8:**
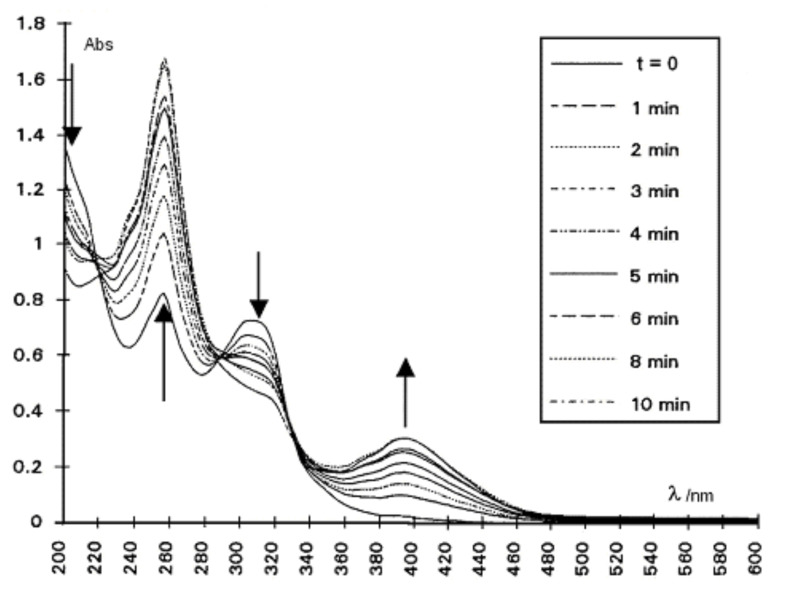
Irradiation at 306 nm of the photoproduct **4** obtained at 400 nm in the same setup; the spectra were recorded at various time intervals, within 10 min, at ambient temperature.

Under the same experimental conditions, the photoproduct **4** was irradiated at 306 nm for 10 min until the starting material spectrum was recovered (Figure 8). Disappearance quantum yield of **4** at 306 nm was found to be 1.6 × 10^−1^ for both solvents.

#### 3.4 Photochromic cycles

**Figure 9:**
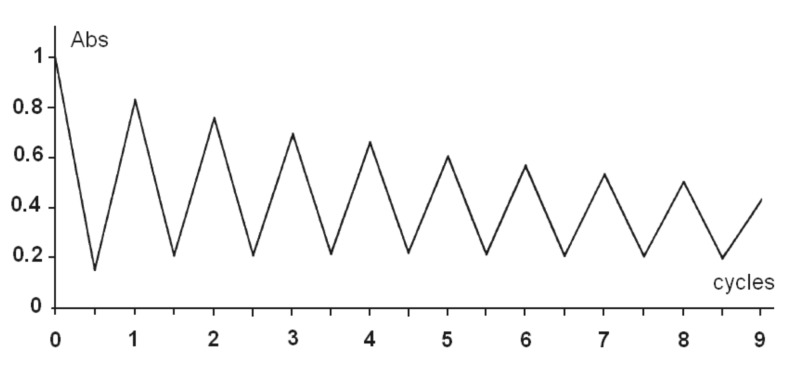
Reversibility of the formation of the photoproduct **4** at 400 nm (40 min) and photodissociation of **4** at 306 nm in MCH.

Using the preceding dilute solution in a quartz cell and the same setup, the medium was alternatively irradiated at 400 nm and at 306 nm in both solvents, and the absorbance measured at 392 nm at each cycle. The results are represented for MCH solution in Figure 9. One observes a drop of 50% absorbance after 8 cycles for MCH and 5 cycles for CH_3_CN (not shown).

The photolysis at 306 nm must involve one or several photoreactions leading to products transparent at 392 nm. Thermal dissociation (a retro Diels-Alder reaction, see below) was expected to exhibit a quantitative yield as shown hereafter. The photoproduct **4**, prepared and isolated as described below, was heated at 55 °C in CDCl_3_. The dissociation was evaluated with time by following the intensity change of the characteristic δ = 4.73 ppm signal (see spectrum below) until complete extinction (see Supporting Information p S2). The dissociation kinetics were found to be first order with a half-life of ca 3.8 h. This suggests a much longer lifetime at ambient temperature, as observed experimentally.

### 4 Structure of the photoproduct

Preparative irradiation of a solution of **2** in CH_2_Cl_2_ through which nitrogen was bubbled with a medium pressure mercury lamp in a Pyrex photoreactor (UV light filtered by NaNO_2_ aqueous solution) gave **4** as a white powder after a week at −20 °C. That a single photoproduct was produced is borne out by HPLC, showing the increase of a single peak at the expense of the starting material (see Supporting Information p S3). No melting point could be determined because the powder decomposes back to **2** on heating. The mass spectrum was found to be that of **2** (see Experimental). Single crystals suitable for X-ray structure analysis could not be obtained. Evidence for the structure rests on the electronic absorption spectrum and the NMR spectral data. The UV spectrum clearly shows the disappearance of the 350–450 nm absorption band, indicating that the anthryl groups have reacted. The new band with a maximum at 306 nm suggests the formation of substituted naphthalenes [21], but because the disubstituted cyclophane also absorbs in that region [22], it is difficult to get more precise information.

**Figure 10:**
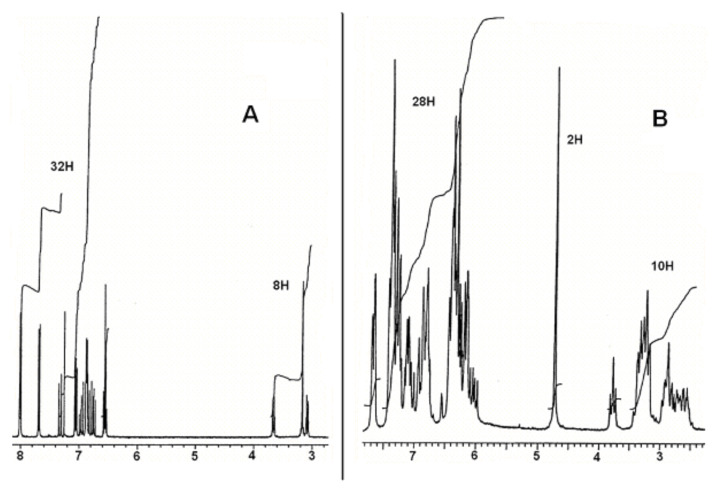
^1^H NMR spectra (400 MHz, CDCl3). A: Compound **2**, B: Compound **4**.

The ^1^H NMR (Figure 10 and Supporting Information p S2) and the ^13^C NMR spectra (52 distinct signals are observed, see Experimental) point to the absence of symmetry in the molecule. This rules out symmetrical cycloadditions such as the 9,9′ : 10,10′ [4+4]reaction of anthracenes [11], whether combined or not with two cyclobutane-forming [2+2]additions. Dissymmetrical closures [11–12] such as 1,4 : 9′,10′ [4+4] or 1,4 : 2′,3′[4+2]cycloadditions (as well as rearranged products) are thus to be considered. A deeper analysis of the experimental data follows.

**^13^C NMR data:** The molecular formula includes 52 carbon atoms; among the 52 signals observed, 16 are due to quaternary C (132 to 142 ppm), 28 to CH (121.3 to 140.4), 4 to other CH likely corresponding to bridgehead carbons (54.9 to 66.0 ppm) and finally 4 to CH_2_ (31.6 to 36.5 ppm), attributable to the ethanocyclophane bridges.

In previously studied cyclophanes, the cyclobutane rings exhibited ^13^C signals between 42 and 51 ppm [18–19]. No such signals are apparent in the present case; therefore it is proposed that no reaction has occurred between the ethylenic bonds. This statement is borne out by the observation of the ^1^H NMR spectrum.

**Figure 11:**
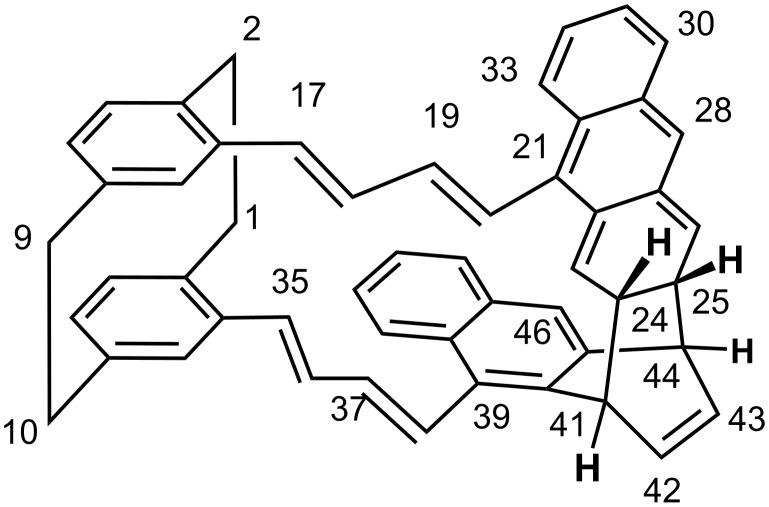
Proposed structure of **4** (1,4 : 2′,3′-cycloadduct).

**^1^H NMR data:** The spectrum exhibits three regions at the following chemical shifts: 7.62–5.97 ppm (28 H) corresponding to aromatic and ethylenic protons; 3.8–2.5 ppm, corresponding to aliphatic protons (10 H) and a prominent signal at 4.73 ppm (2 H, broad singlet) attributable also to aliphatic protons. Compared to **2**, there are four new signals corresponding to aliphatic protons, in keeping with the cycloadduct pictured in Figure 11.

This is a Diels-Alder adduct resulting from the reaction of the 1,4 positions (C41 and C44) of one nucleus and the 2′,3′ positions of the other nucleus (C24 and C25).The structure of **4** is further supported by the ^1^H-^1^H COSY spectrum (see Supporting Information p S4).

It indicates that the signal at δ = 3.8 (pseudotriplet) is coupled with that at δ = 3.2 and that at δ = 6.05, whereas the signal at 3.2 is coupled with those at δ = 3.8 and 6.38 ppm, respectively. The signals at 3.2 and 3.8 ppm can be attributed to allylic bridgehead H24 and H25, coupled with the vicinal ethylenic hydrogen atoms (23 and 26) at 6.05 and 6.38 ppm. Bridgehead protons H41 and H44 then display the peak at δ = 4.73 ppm, reflecting a very small coupling with their neighbours (H–C–C–H dihedral angles close to 90°) [23–24].

The majority of anthracene derivatives are known to undergo a [4+4]cycloaddition from the S_1_ state [11–12], but some [4+2]-cycloadditions were observed to occur under geometrical constraints [11–12,24] through a triplet state process [25]. [4+2]-Cycloadditions can take place in a hot ground state [26]. This result is reminiscent of the [4+2]-photocycloaddition between two naphthalene substrates for the anti-[2.2](1,4)-naphthalenophane leading to dibenzoequinine, a stable polycyclic molecule [27]. In that case, the first Diels-Alder addition is immediately followed by a second, because of the superimposition of the newly formed diene and the ene centres (cascade reaction). This is not possible in the present situation, considering Figure 11. The non-reactivity of the ethylenic bonds is understandable as the reaction affects first the anthracene substrates leading to **4** in which the double bonds are no longer at mutual distances conducive to further reactions. Such a 1,4 : 2′,3′-cyclophotoaddition between two anthracene nuclei is unprecedented.

## Summary and Conclusion

The target molecule (dianthryl-butadienyl[2.2]paracyclophane **2**) was synthesized and shown to possess the anticipated photochromic properties. The two interconverting forms exhibit good thermal stability. The cycloreversion can be induced by irradiation or by heating. Because of its slow response and the fatigue observed in the photodissociation, this system does not seem suitable as a switch, but might be considered for applications such as optical storage [28], owing to the large spectral shift (Δ
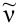
_max_ ca 7500 cm^−1^) of the electronic absorption spectra between **2** (λ_max_ 400 nm) and **4** (λ_max_ 306 nm) and the stability of the photoproduct in interior daylight.

## Experimental

### General Techniques

Melting Points (up to 200 °C) were taken with a Büchi 510 Melting Point Appararus. Those above 200 °C were measured with a Kofler Heiztischmikroskop Thermopan (Reichert); all m.p. are uncorrected. HPLC was performed using a Nucleosil 100 −7 octadecyl phase (Macherey Nagel, Düren, No 715802) column, with a pump L-6200 and a photodiode array detector L-3000 (Hitachi). Column chromatography: Merck Kieselgel 60 (70–230 mesh). Thin layer chromatography: Kieselgel (Merck) or Alox (Macherey-Nagel). Elemental Analyses were obtained by the Institute of Inorganic and Analytical Chemistry and of Pharmaceutical Chemistry of the Technical University of Braunschweig.

### Spectrometry

NMR spectra were recorded with an AC-200 (^1^H: 200 MHz, ^13^C: 50 MHz) or an AM-400 (^1^H: 400 MHz, ^13^C: 100 MHz) Bruker apparatus. The chemical shifts were measured with tetramethylsilane as internal reference. The ^1^H-^1^H COSY spectrum was used to interpret the spectrum of **4**, and the DEPT technique to assign the class of carbon atoms in the ^13^C spectra. The IR spectra were recorded with a Nicolet 320 FT spectrometer. Mass spectra were performed with a Finnigan MAT 8430 spectrometer using the classical Electron Ionization at 70 eV or the FAB technique, respectively. Electronic absorption spectra were recorded with a HP 8542 A-Diode Array or a Hitachi UV 3300 spectrometer. The samples were weighed with a Mettler UM3 balance (sensitivity 10^−7^ g).

### Quantum yields

Reaction quantum yields were determined as described elsewhere [29], using the Parker iron trioxalate actinometer. The monochromatic beams were obtained from a cooled 1000 W Xenon lamp, using a Bausch and Lomb monochromator. The samples were previously purged of oxygen with an argon or nitrogen stream.

### Preparations

1. 4,13-bis[(1*E*,3*E*)-4-(9-anthracenyl)-buta-1,3-dienyl][2.2]paracyclophane (**2**):

In a 250 mL, dried round-bottom flask, equipped with a reflux condenser, a Claisen adapter, a stirring system, and degassed with nitrogen, 1.3 g dialdehyde (4.1 mmol) was dissolved in 50 mL absolute THF. In another vessel, 6.56 g (12.3 mmol) of 9-anthryltriphenylphosphoniumbromide, in suspension in 50 mL THF under nitrogen, was mixed with a solution of 8.2 mL BuLi (1.5 M, hexane). The resulting deep red solution was introduced dropwise with a needle into the dialdehyde solution and the reaction medium was stirred overnight at ambient temperature. Finally, the reaction mixture was hydrolyzed with crushed ice/water. The crude product was filtered off, dissolved in a small volume of CH_2_Cl_2_ and dried over MgSO_4_. After adding 3 g of silica gel to the filtered solution, the solvent was distilled off under reduced pressure and the residue was eluted on a 100 g silica gel column with 1.5 L of pentane and then a pentane/CH_2_Cl_2_: 4/1 mixture. Compound **2** (1.83 g) was obtained as a mixture of *cis*/*trans* isomers. The solid was dissolved in about 200 mL of CH_2_Cl_2_ in an ultrasonic bath, with gentle warming. To this solution was added about 500 mL of pentane and the flask was cooled to −20 °C. After two days, a precipitate of fine crystals appeared, which were filtered off and carefully washed with pentane. After drying under high vacuum, 1.32 g (1.99 mmol, 48% yield) of all-*trans* **2** was isolated as an orange microcrystalline powder. – R_f_ (SiO_2_; pentane/CH_2_Cl_2_: 2/1): 0.41; mp 217 °C; – ^1^H-NMR (400 MHz, CDCl_3_, 25 °C, TMS): δ = 8.00 (s, 2 H; 28-H, 46-H), 8.00 (br-d, ^3^*J*(H,H) = 8.4 Hz, 4 H; 26-H, 30-H, 44-H, 48-H), 7.68 (br-d, ^3^*J*(H,H) = 8.4 Hz, 4 H; 23-H, 33-H, 41-H, 51-H), 7.32 (d, ^3^*J*(H,H) = 15.7 Hz, 2 H; 20-H, 38-H), 7.05 (br-ps-t, ^3^*J*(H,H) = 8.4 Hz, 4 H, 24-H, 32-H, 42-H, 50-H), 6.96 (dd, ^3^*J*(H,H) = 15.3 Hz, ^3^*J*(H,H) = 10.4 Hz, 2 H; 18-H, 36-H), 6.87–6.81 (m, 8 H; 5-H, 12-H, 17-H, 35-H, 25-H, 31-H, 43-H, 49-H), 6.75 (dd, ^3^*J*(H,H) = 15.7 Hz, ^3^*J*(H,H) = 10.4 Hz, 2 H; 19-H, 37-H), 6.57 (d, ^3^*J*(H,H) = 7.8 Hz, 2 H; 8-H, 15-H), 6.53 (dd, ^3^*J*(H,H) = 7.8 Hz, ^4^*J*(H,H) = 1.7 Hz, 2 H ; 7-H, 16-H), 3.67–3.64 (m, 2 H ; 1a-H, 2a-H), 3.17 (s, 4 H ; 9-H, 10-H), 3.09–3.06 ppm (m, 2 H ; 1b-H, 2b-H); ^13^C-NMR (100 MHz, CDCl_3_, 25 °C, TMS): δ = 139.5 (qC), 138.1 (CH), 137.7 (qC), 134.9 (CH), 132.3 (CH), 131.7 (CH), 131.1 (qC), 130.1 (CH), 129.8 (CH), 129.0 (qC), 128.2 (CH), 126.0 (CH), 125.3 (CH), 124.8 (CH), 124.5 (CH), 35.1 (CH_2_), 32.8 ppm (CH_2_); – MS (70 eV): *m/z* (%) = 664 (78) [M^+^], 473 (15) [M^+^-191], 332 (23) [C_8_H_7_CHCHCHCH-A^+^], 331 (43) [C_26_H_19_^+^], 315 (24), 215 (25), 203 (34), 191 (100) [A-CH_2_^+^]; – UV/Vis (CH_3_CN): λ_max_ (lg *ε*) = 256 (4.96), 320 (sh) (4.23), 372 (sh) (4.07), 392 nm (4.19); – UV/Vis (methylcyclohexane): λ_max_ (lg *ε*) = 218 (sh) (4.71), 240 (sh) (4.90), 258 (5.07), 316 (4.38), 396 nm (4.36); – IR (KBr): 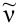 = 3019 cm^−1^ (m), 2923 (m), 1621 (w), 1441 (w), 1407 (w), 994 (s), 950 (m), 880 (s), 839 (m), 779 (m), 730 (vs), 717 (m); – HRMS (FAB): *m/z* calcd: 664.313001, found: 664.313 ± 3 ppm; Elemental analysis: calcd C 93.94, H 6.06, found C 91.74, H 6.03.

2. Photoproduct **4**: A solution of 133 mg (0.2 mmol) of **2** in 200 mL of CH_2_Cl_2_, carefully degassed by bubbling nitrogen through it, was irradiated at λ > 400 nm for 2 h with a high pressure mercury lamp in a Pyrex photoreactor. The cooling tube of the latter was filled with a NaNO_2_ solution (75 g/L) maintaining the reaction medium at 10–20 °C. After the end of irradiation, the solution was concentrated at ca 15 °C to 10 mL; then 25 mL pentane was added and the medium allowed to stand at −20 °C for a week. A white precipitate appeared, which was filtered off and washed with pentane. After desiccation under high vacuum, **4** was isolated (71 mg: 0.11 mmol, 53%). From the mother liquor, some additional powder was collected and identified as the starting material **2**. The irradiation was followed by HPLC monitoring after 30, 90, and 120 min (see Supporting Information p S3). R_f_ (Alox; CH_2_Cl_2_): 0.49. mp. dec. by heating; ^1^H-NMR (400 MHz, CDCl_3_, 25 °C, TMS): δ = 7.62–7.58 (m, 2 H), 7.38–7.17 (m, 8 H), 7.08–6.95 (m, 2 H), 6.93–6.67 (m, 4 H), 6.40–6.20 (m, 9 H), 6.13 (dd, ^3^*J*(H,H) = 7.6 Hz, ^3^*J*(H,H) = 11.8 Hz, 1 H), 6.08 (br-s, 1 H), 5.97 (dd, ^3^*J*(H,H) = 8.7 Hz, ^3^*J*(H,H) = 14.8 Hz, 1 H) 4.65 (br-s, 2 H), 3.70 (ps-t, ^3^*J*(H,H) = 8.3 Hz, 1 H), 3.32–3.08 (m, 5 H), 2.87–2.75 ppm (m, 4 H); – ^13^C-NMR (100 MHz, CDCl_3_, 25 °C, TMS): δ = 142.0 (qC), 141.6 (qC), 140.5 (qC), 140.4 (CH), 139.9 (qC), 139.4 (qC), 139.2 (qC), 138.6 (qC), 137.6 (qC), 137.1 (qC), 137.0 (qC), 136.6 (qC), 136.1 (CH), 136.0 (qC), 135.8 (qC), 135.71 (qC), 135.67 (qC), 135.6 (CH), 135.0 (CH), 134.3 (CH), 133.3 (CH), 132.0 (qC), 131.5 (CH), 130.8 (CH), 130.5 (CH), 129.9 (CH), 129.8 (CH), 128.6 (CH), 128.32 (CH), 128.28 (CH), 128.2 (CH), 126.7 (CH), 126.5 (CH), 125.4 (CH), 125.35 (CH), 125.32 (CH), 125.1 (CH), 125.0 (CH), 124.8 (CH), 124.5 (CH), 124.0 (CH), 123.9 (CH), 121.5 (CH), 121.3 (CH), 66.0 (CH), 56.2 (CH), 55.0 (CH), 54.9 (CH), 36.5 (CH_2_), 36.3 (CH_2_), 35.4 (CH_2_), 31.6 ppm (CH_2_). The mass spectrum was found to be identical to that of compound **2**.

3. X-Ray structure determination of **2**·1/2CH_2_Cl_2_

*Crystal data*: C_52.5_H_41_Cl, Mr = 707.30, monoclinic, P2_1_/c, T = −100 °C, a = 17.716(3), b = 10.236(2), c = 21.811(3) Å, β = 110.523(8)°, U = 3704.1 Å^3^, Z = 4, F(000) = 1492, λ (Mo K_α_) = 0.71073 Å, μ = 0.14 mm^−1^, D_x_ = 1.268 g cm^−3^. *Data collection*: A yellow lath ca. 0.9 × 0.2 × 0.1 mm was mounted in inert oil on a glass fibre and transferred to the cold gas stream of a Siemens P4 diffractometer. Data were recorded to 2θ 50°. *Structure refinement*: The structure was refined using SHELXL-97 [30]. Hydrogen atoms were included using a riding model. The dichloromethane molecule is disordered across an inversion centre. Restraints to light atom *U* values were applied. The final wR2 (all reflections) was 0.124 for 6369 intensities, 491 parameters and 516 restraints, with R1 (I>2σ(I)) 0.052; S 0.77, max. Δρ 0.31 e Å^−3^. See also Supporting Information p S5.

X-ray crystallographic data (excluding structure factors) were deposited under the number CCDC-717774 and can be obtained free of charge from the Cambridge Crystallographic Data Centre via www.ccdc.cam.ac.uk/data_request/cif.

## Supporting Information

File 1 Thermal dissociation of photoproduct **4**, HPLC diagram of the photochemical preparation of **4**, ^1^H-^1^H-COSY spectrum of photoproduct **4**, and other crystal data for **2**.
