# Supplementary material for: Reversible intramolecular photocycloaddition of a bis(9-anthrylbutadienyl)paracyclophane – an inverse photochromic system. (Photoactive cyclophanes 5)
Source: Beilstein J Org Chem. 2009 May 7;5:20. doi: 10.3762/bjoc.5.20 (PMC2707062; doi:10.3762/bjoc.5.20)
Supplement: File 1 — Thermal dissociation of photoproduct 4, HPLC diagram of the photochemical preparation of 4, 1H-1H-COSY spectrum of photoproduct 4, and other crystal data for 2. [file Beilstein_J_Org_Chem-05-20-s001.doc]

**Supporting information for:**

**Reversible intramolecular photocycloaddition of a bis(9-anthrylbutadienyl)paracyclophane – an inverse photochromic system. (Photoactive cyclophanes 5)**

Henning Hopf,*^1^ Christian Beck,^1^ Jean-Pierre Desvergne,^2^ Henri Bouas-Laurent,*^2^ Peter G. Jones^3^, and Ludger Ernst^4^.

Address: ^1^Institut für Organische Chemie, Technische Universität Braunschweig, Postfach 3329, D-38023 Braunschweig, Germany, ^2^ISM, CNRS UMR 5255, Université Bordeaux 1, F- 33405 Talence Cedex, France, ^3^Institut für Anorganische Chemie, Technische Universität Braunschweig, Postfach 3329, D- 38023 Braunschweig, Germany and ^4^NMR-Laboratorium der Chemischen Institute der Technischen Universität Braunschweig, Hagenring 30, D-38106 Braunschweig, Germany

Email: H. Hopf* - h.hopf@tu-bs.de; Jean-Pierre Desvergne - jp.desvergne@ism.u-bordeaux1.fr; H. Bouas-Laurent* - h.bouaslaurent@cegetel.net; Peter. G. Jones - p.jones@tu-bs.de; L. Ernst - l.ernst@tu-bs.de

^*^ Corresponding author

**Table of contents:**

S2: Thermal dissociation of photoproduct **4**

S3: HPLC diagram of the photochemical preparation of **4**

S4: ^1^H-^1^H-COSY spectrum of photoproduct **4**

S5: other crystal data for **2**


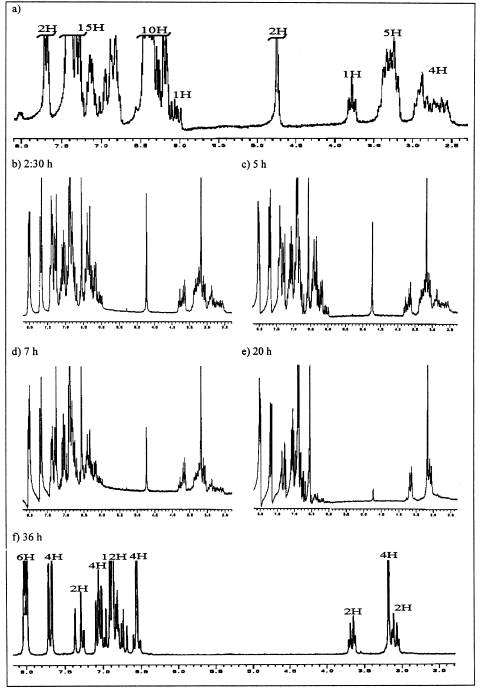


Thermal dissociation of photoproduct **4** in CDCl_3_ at 55 °C. The kinetics was followed by the intensity decrease of the δ = 4.73 ppm signal, which was completed after 36 h without apparent degradation of the material.

**
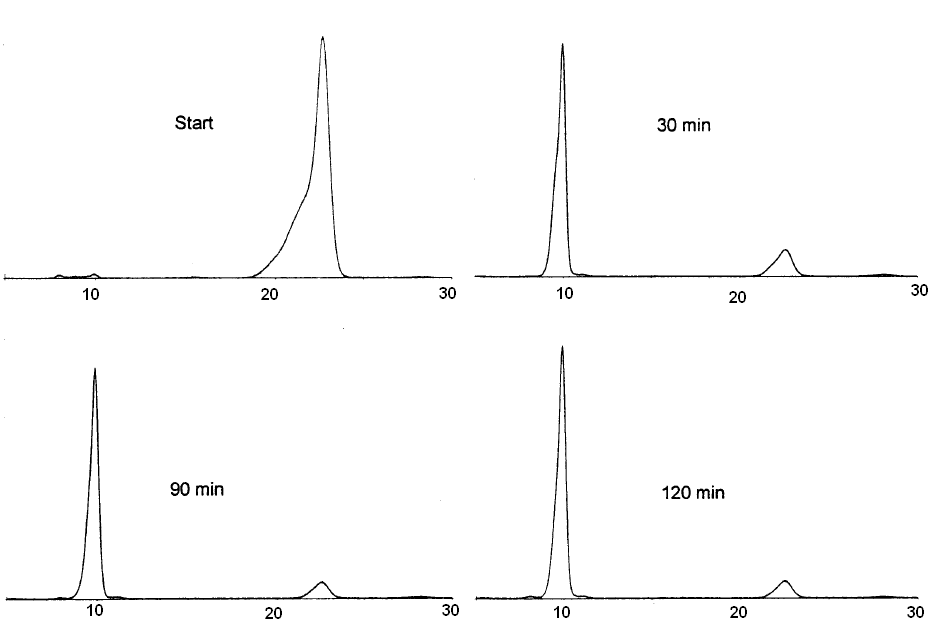
**

HPLC diagram of the photochemical transformation of **2** (right signal) into **4** (left signal), as a function of irradiation time, under preparative conditions (see experimental). UV detection: 290 nm

**
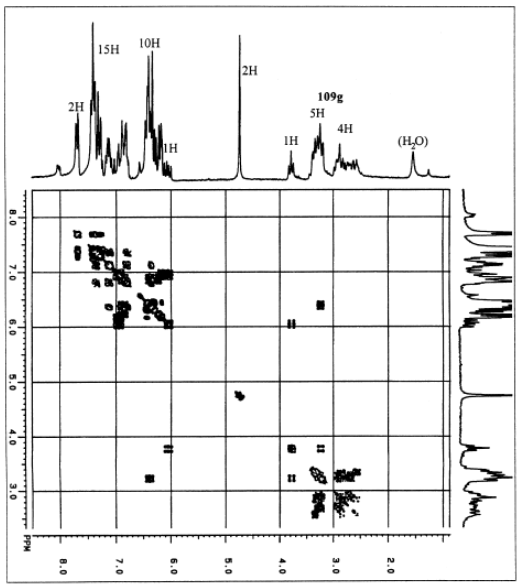
**

^1^H-^1^H-COSY spectrum of photoproduct **4**

**Bond precision: C-C = 0.0048 A Wavelength=0.71073**

**Cell: a=17.716(3) b=10.236(2) c=21.811(3)**

**alpha=90 beta=110.523(8) gamma=90**

**Temperature: 173 K**

**Calculated Reported**

**Volume 3704.2(11) 3704.1(11)**

**Space group P 21/c P 21/c**

**Hall group -P 2ybc ?**

**Moiety formula 2(C52 H40), C H2 Cl2 ?**

**Sum formula C105 H82 Cl2 C52.50 H41 CL**

**Mr 1414.61 707.30**

**Dx,g cm-3 1.268 1.268**

**Z 2 4**

**Mu (mm-1) 0.141 0.141**

**F000 1492.0 1492.0**

**F000’ 1493.11**

**h,k,lmax 21,12,25 21,12,24**

**Nref 6522 6369**

**Tmin,Tmax 0.967,0.986**

**Tmin’ 0.881**

**Correction method= Not given**

**Data completeness= 0.977 Theta(max)= 25.000**

**R(reflections)= 0.0522( 2592) wR2(reflections)= 0.1244( 6369)**

**S = 1.043 Npar= 491**
